# Evaluation of Paediatric Melanoma Management in a UK Major Tertiary Centre: A Twenty-Year Review

**DOI:** 10.7759/cureus.89649

**Published:** 2025-08-08

**Authors:** Amit Nijran, Kin Seng Tong, Pavandeep Kaur Virdee, Jagajeevan Jagadeesan, Ramesh Vidyadharan

**Affiliations:** 1 Plastic and Reconstructive Surgery, Birmingham Women’s and Children’s NHS Foundation Trust, Birmingham, GBR; 2 Trauma and Orthopaedics, Sandwell and West Birmingham Hospitals NHS Trust, Birmingham, GBR

**Keywords:** diagnosis, evidence, guidelines, management, melanoma, paediatric, paediatric melanoma, plastic surgery, treatment

## Abstract

Objectives: This study evaluates the management of paediatric melanoma at a tertiary centre, comparing clinical practices with international guidelines from the European Cooperative Study Group for Pediatric Rare Tumors (EXPeRT) and the National Comprehensive Cancer Network (NCCN) to highlight real-world practices and make recommendations for future research priorities. The differences between conventional and Spitzoid melanomas were also explored in a subgroup analysis.

Background: Paediatric melanoma is rare and is most commonly caused by UV exposure or familial mutations. Management of paediatric melanoma is challenging due to diagnostic, histological and treatment complexities, coupled with limited paediatric-specific evidence. Treatment typically involves wide local excision (WLE) for localised melanoma. Advanced cases may require systemic therapy, such as chemotherapy or immunotherapy; however, paediatric-specific clinical trials to support treatment protocols are limited, and therefore, treatment is initiated on a case-by-case basis.

Methods: A retrospective case series analysis was completed for all melanoma patients aged between 0 and 15 years presenting at Birmingham Children’s Hospital, a UK tertiary paediatric centre, between 2003 and 2023. Statistical analysis was performed using the t-test for continuous variables and Fisher’s exact test for categorical data.

Results: Eight cases were treated, with an average diagnosis age of 9.8 years. Tumours were most often located on the extremities (4/8, 50%), followed by the head and neck (2/8, 25%). All cases underwent WLE, and three (3/8, 38%) were considered for sentinel lymph node biopsy (SLNB). There was 100% (8/8) compliance with discussion in a specialist melanoma multidisciplinary team (MDT) and review by experienced dermatopathologists. Follow-up imaging and compliance with guidelines were inconsistent, with only two (2/8, 25%) receiving recommended imaging.

Conclusions: Findings show excellent compliance with key recommendations that all cases should be discussed in a specialist melanoma MDT and histology reviewed by an experienced dermatopathologist. There was variability in biopsy techniques, imaging, and follow-up, suggesting a need for standardised guidelines to guide best practice. Future research should include multicentre, randomised, blinded paediatric-specific clinical trials to compare outcomes to potential treatments.

## Introduction

Paediatric melanoma is rare, accounting for 1% of all paediatric malignancies [1] and commonly affecting the head and neck region [2,3]. Sporadic DNA changes due to UV exposure account for the majority of paediatric melanoma, whilst 22% arise from a range of non-modifiable risk factors, including familial mutations, number of naevi and radiation exposure [4].

The diagnosis of paediatric melanoma is challenging, as morphological appearance may not correspond to biological behaviours. For instance, benign tumours such as Spitz naevi and atypical Spitz naevi may mimic melanoma both clinically and microscopically [5-7]. The ABCDE rule, used to identify suspected melanomas based on superficial appearances in adults, does not apply to certain paediatric presentations, such as amelanotic lesions and malignant transformation of congenital naevi [8,9]. Furthermore, if melanoma develops within a congenital melanocytic naevus, it often involves the deeper component rather than the peripheral component, which can result in a delayed diagnosis [2].

There are also differences in subtypes and mutation patterns in paediatric melanoma compared to adult melanoma, such as the relatively higher incidence of Spitzoid melanomas and the relative lack of BRAF V600E and TERT promoter mutations [4]. Spitzoid melanomas often have kinase fusion gene alterations such as ALK, NTRK and ROS1, but when TERT promoter mutations are found, they may be a marker of a more aggressive behaviour [4]. Common adult prognostic markers should be interpreted with caution, due to cases where Spitzoid melanoma exhibits a slower growth course despite higher Breslow thickness (BT) and higher propensity for a positive sentinel lymph node biopsy (SLNB) [1,10].

In addition to the difficulties in diagnosis, treatment strategies in paediatric melanoma are often extrapolated from adult data despite differences in pharmacokinetic profile and pathological mechanism [9]. Most paediatric melanomas are localised (melanoma in situ or stages I and II) and are treated with wide local excision (WLE), with or without SLNB [11]. Advanced disease (stages III and IV) carries a poor prognosis, requiring surgery and targeted therapies, including chemotherapy and immunotherapy [12]. Whilst the treatment efficacy of targeted agents has been demonstrated in the adult population, clinical trials involving children have been terminated prematurely due to low recruitment [1]. Moreover, no current clinical guidance exists with regard to monitoring for the recurrence of paediatric melanoma at the time of writing.

The incidence of paediatric melanoma varies amongst pre-adolescent (<15 years old) and adolescent (15-19 years old) populations, with incidence rates of 1.1 per million and 10.4 per million, respectively [13]. The higher incidence in adolescent populations may be explained by increased cumulative UV exposure [14,15]. The combination of both age groups, however, results in an overall incidence of paediatric melanoma of 4.5 per million population, which excludes it from being classed as a 'very rare tumour' as defined by leading paediatric rare tumour research networks, such as the European Cooperative Study Group for Pediatric Rare Tumors (EXPeRT) and the European Union Joint Action on Rare Cancers (JARC) [2]. This precludes paediatric melanoma from the same clinical expertise and priority as other very rare paediatric malignancies.

The differences in pathophysiology and presentation of paediatric melanoma and the lack of paediatric-specific research and guidelines present challenges for clinicians in their diagnosis and management of paediatric melanoma [13]. In this study, we evaluated the management of eight cases of paediatric melanoma at a single tertiary centre over a 20-year period, comparing this against the recommendations from two leading international melanoma research networks: the EXPeRT [9] and the National Comprehensive Cancer Network (NCCN) [4]. As a secondary aim, we conducted an exploratory subgroup analysis to compare patients with conventional melanoma and Spitzoid melanoma, given their biological and histological differences and the consequent treatment implications.

## Materials and methods

Study design

A retrospective case series analysis was conducted to evaluate diagnostic pathways, treatment modalities, and follow-up strategies for paediatric melanoma. The study compared real-world clinical practice with evidence-based international guidelines, specifically the EXPeRT and the NCCN recommendations [4,9]. These guidelines have been chosen as the NCCN guidelines are updated annually and are more widely used internationally and therefore provide a consistent comparison against contemporary research in the field, despite being adult-specific. The EXPeRT guidelines provide the only paediatric-specific recommendation during the research period. The Children’s Oncology Group produced paediatric-specific guidelines in late 2023; however, this was outside the study timeline and therefore not considered for comparison [16]. We also performed an exploratory subgroup analysis comparing conventional and Spitzoid melanomas due to their biological and histological differences and consequent treatment implications.

Study timeline

The study reviewed paediatric melanoma cases diagnosed and managed between January 1, 2003, and December 31, 2023.

Study population

Cases were identified through the hospital’s histopathology system using Systematized Nomenclature of Medicine (SNOMED) codes corresponding to ‘melanoma’ and ‘malignant melanoma’ [17]. These were cross-referenced with the Somerset Cancer Register, a national UK cancer database, to ensure comprehensive identification of eligible cases [18]. The cohort included all patients aged 0-15 years who were diagnosed with melanoma and received treatment at Birmingham Children’s Hospital during the study period. Local arrangements meant that children and young people aged 16-18 transitioned to adult services; therefore, this age group was not included in this study.

Study measures

Clinical data were extracted from patient records, including electronic and paper-based health records, histopathology reports, surgical notes, and outpatient clinic documentation. Variables assessed included demographic data, clinical presentation, histopathological diagnosis, MDT decision-making, surgical interventions (including biopsy and excision margins), use of SLNB, imaging for staging or surveillance, adjuvant therapy, and follow-up frequency. Each case was evaluated for its alignment with guideline-based best practices from EXPeRT and NCCN.

Inclusion and exclusion criteria

Inclusion criteria of the study were patients aged between 0 and 15 years at the time of melanoma diagnosis, with histologically confirmed diagnosis of melanoma or malignant melanoma, and who received treatment at Birmingham Children’s Hospital during the study period.

Exclusion criteria were patients with melanocytic lesions not meeting diagnostic criteria for melanoma.

Ethics statement

The study was conducted in accordance with the principles of the Declaration of Helsinki. As a retrospective study using anonymised data, individual patient consent was waived.

Statistical analysis

Descriptive statistics were used to summarise demographic and clinical characteristics. The Shapiro-Wilk test was performed to test for normality; normally distributed data were presented using mean and standard deviation (SD), while skewed data were presented using median and interquartile range (IQR). Continuous variables were compared using the two-tailed Student’s t-test for parametric data or the Mann-Whitney U test for skewed data, and categorical variables were analysed using Fisher’s exact test due to the relatively small sample size. A p-value of <0.05 was considered statistically significant. Statistical analysis was performed using GraphPad Prism version 10.0.0 for Windows (Dotmatics, Boston, MA, US).

## Results

Over a 20-year period, a total of eight patients with primary cutaneous melanoma were treated at Birmingham Children’s Hospital (Table 1). There were four males and four females with a mean age of 9.8 years at diagnosis (range 4-15). There was no documentation of significant risk factors related to melanoma, including Fitzpatrick skin type, history of sun exposure and immunosuppression. Two patients had a family history of melanoma (first-degree and third-degree relatives); however, this did not meet the criteria for genetic testing for genodermatoses. No patients developed melanoma arising from a congenital melanocytic naevus. The average time from lesion onset to diagnosis was 7.9 months (range 3-17), with four cases having their initial biopsy performed by the plastic surgery team. The remaining four patients were biopsied by dermatologists and general practitioners (primary care physicians). Biopsy procedures included curettage and cautery, shave biopsy and excision biopsy, with no association between the type of biopsy and the specialty performing the procedure (p = 0.31). There was also no association between the type of biopsy (curettage and cautery/shave biopsy vs excision biopsy) and the time to diagnosis (p = 0.78). The extremities were the most common site of melanoma (4/8, 50%), followed by the head and neck (2/8, 25%) and truncal regions (2/8, 25%).

**Table 1:** Characteristics of patients with paediatric cutaneous melanoma The Shapiro-Wilk test was performed to test for normality for age at diagnosis (p = 0.86), time to diagnosis (p = 0.08) and Breslow thickness (p = 0.42). *Values indicate the number of patients (%), unless otherwise indicated.

Characteristic	No. (%)*
Age at diagnosis (years)	-
Mean (SD)	9.8 (3.8)
Sex	-
Female	4 (50)
Male	4 (50)
Family history of melanoma	-
Yes	2 (25)
No	6 (75)
Time to diagnosis (months)	-
Mean (SD)	7.9 (5.5)
Specialty performing biopsy	-
Primary care	2 (25)
Dermatology	2 (25)
Plastic surgery	4 (50)
Primary tumour location	-
Extremity	4 (50)
Head or neck	2 (25)
Trunk	2 (25)
Histology subtypes	-
Superficial spreading	2 (25)
Nodular	3 (37.5)
Spitzoid	2 (25)
Non-classified	1 (12.5)
Breslow thickness (mm)	-
Mean (SD)	6.8 (4.6)
Tumour depth	-
T1 (≤1.0 mm)	0 (0)
T2 (>1.0 to 2.0 mm)	1 (12.5)
T3 (>2.0 to 4.0 mm)	3 (37.5)
T4 (>4.0 mm)	4 (50)

All histology reports provided information on BT, but data on ulceration were incomplete. There was a near-equal distribution in the histological subtypes of melanoma, including superficial spreading, nodular and Spitzoid. When diagnostic uncertainty arose, molecular testing was performed in six cases, for example, testing for MIB-1 to distinguish from Spitz naevus, HMB45 to differentiate from atypical Spitz tumour and S100 to identify spindle cell melanoma from atypical spindle cell tumour. The mean BT reported was 6.8 mm, with a range of 1.5-14 mm. Half of the cases presented with a histopathological T4 stage, but there was no significant association between the T staging and the histological subtype (p = 1.00) nor the patient’s age at diagnosis (p = 0.14).

All cases were discussed in a specialist multidisciplinary team (MDT) setting with histological slides reviewed by experienced dermatopathologists and a consensus plan agreed upon (Table 2). Definitive management involved WLE of the melanoma scar in all cases. In two patients, additional procedures were performed due to clinically palpable lymphadenopathy. SLNB was considered in three cases but decided against by the MDT in view of the patients’ young ages, the significant morbidity associated with a procedure that only provides prognostic and not a therapeutic benefit, and the fact that adjuvant therapies were not licensed for paediatric use.

**Table 2:** Diagnosis and definitive management WLE: wide local excision, SLNB: sentinel lymph node biopsy, N/A: not applicable.

Characteristic	No. (%)
Dermoscopy performed	-
Yes	1 (12.5)
No	7 (87.5)
Diagnostic uncertainty	-
Yes	4 (50)
No	4 (50)
Molecular testing	-
Yes	6 (75)
No	1 (12.5)
N/A	1 (12.5)
Definitive management	-
WLE	8 (100)
Additional procedures	-
Inguinal lymphadenectomy	1 (12.5)
Open biopsy of supraclavicular node	1 (12.5)
Excision margin	-
1 cm	1 (12.5)
1.1-2 cm	4 (50)
2.1-3 cm	3 (37.5)
SLNB considered	-
Yes	3 (37.5)
No	3 (37.5)
N/A	2 (25)

The period of follow-up ranged from three months to over five years (Table 3). In terms of interval radiological surveillance, two patients received all the appropriate scans in line with the EXPeRT recommendations, three patients received some of the recommended scans, while three patients did not undergo the appropriate imaging surveillance. One patient had a recurrence of melanoma 29 months after WLE, with metastasis to the ipsilateral cervical nodes and contralateral thigh and lung. The patient was treated with nivolumab off license. All patients were alive at the end of the study timeline.

**Table 3:** Follow-up regime and outcomes

Characteristic	No. (%)
Imaging in line with recommendation	-
Yes	2 (25)
Partial	3 (37.5)
No	3 (37.5)
Recurrence	-
Yes	1 (12.5)
No	7 (87.5)
Metastasis	-
Yes	1 (12.5)
No	7 (87.5)

A subgroup analysis comparing patient characteristics and treatments for conventional melanoma versus Spitzoid melanoma did not yield any positive findings between the two categories of patients (Table 4).

**Table 4:** Comparison between conventional melanoma and Spitzoid melanoma The Shapiro-Wilk test was performed to test for normality for age at diagnosis (p = 0.85), Breslow thickness (p = 0.21) and follow-up duration (p = 0.72). *Values indicate the number of patients (%), unless otherwise indicated.

Characteristic	Conventional melanoma (n = 6), n (%)*	Spitzoid melanoma (n = 2), n (%)*	p-value (t-statistic)	Fisher’s exact test
Age at diagnosis (years)			0.06 (2.90)	
Mean (SD)	11.2 (3.1)	5.5 (2.1)		
Age at diagnosis (years)				0.11
<10	1 (17)	2 (100)		
≥10	5 (83)	0 (0)		
Primary tumour location				0.07
Extremity	4 (67)	0 (0)		
Head or neck	2 (33)	0 (0)		
Trunk	0 (0)	2 (100)		
Breslow thickness (mm)			0.15 (1.62)	
Mean (SD)	5.4 (4.2)	11 (4.2)		
Tumour depth				0.57
T1 (≤1.0 mm)	0 (0)	0 (0)		
T2 (>1.0 to 2.0 mm)	1 (17)	0 (0)		
T3 (>2.0 to 4.0 mm)	3 (50)	0 (0)		
T4 (>4.0 mm)	2 (33)	2 (100)		
Surgical margins				0.36
0–1 cm	0 (0)	1 (50)		
1.1–2 cm	3 (50)	1 (50)		
2.1–3 cm	3 (50)	0 (0)		
Adjuvant treatment				1.00
Yes	1 (17)	0 (0)		
No	5 (83)	2 (100)		
Recurrence				1.00
Yes	1 (17)	0 (0)		
No	5 (83)	2 (100)		
Metastasis				1.00
Yes	2 (33)	1 (50)		
No	4 (67)	1 (50)		
Follow-up (months)			0.12 (2.01)	
Mean (SD)	27.8 (21.9)	52 (11.3)		
Imaging in line with recommendations				0.25
Yes	2 (33)	0 (0)		
Partial	1 (17)	2 (100)		
No	3 (50)	0 (0)		

## Discussion

The results indicate an inconsistent diagnostic, management and follow-up pathway for the treatment of paediatric melanoma (Table 5). Over the 20-year analysis period, eight patients were diagnosed with cutaneous melanoma. The time to diagnosis ranged from 3 to 17 months with a mean time of 7.9 months. Cordoro et al. [12] reported similar findings in their study, which showed that 82% (58/70) of diagnoses took more than six months from initial detection, and postulated that this may have been due to the atypical presentation of paediatric melanoma, which does not follow the ABCDE criteria used for clinical detection in adults. This is significantly longer than the ‘faster diagnosis target’ introduced by NHS England in 2023, which aims to ensure that 75% of patients with suspected cancer achieve a timely diagnosis within 28 days [19].

**Table 5:** Recommendations from EXPeRT and NCCN papers and compliance of our study sample and areas of possible future research/guidelines

Recommendation from EXPeRT + NCCN	Compliance	Reasons for non-compliance	Recommendations for future research/guidelines
Excision biopsy with at least 2 mm peripheral margin	75% (6/8)	Difficulty in diagnosis and awareness of paediatric melanoma	National Paediatric Melanoma Guidelines
Histology reviewed by experienced dermatopathologists	100% (8/8)	N/A	N/A
MDT discussion	100% (8/8)	N/A	N/A
SLNB for BT > 0.8 mm pT1b	38% (3/8)	As adjuvant therapy only used for stage III disease due to lack of evidence in paediatric population, children not offered SLNB for stage II or below	Robust clinical trials involving children and adjuvant therapy
Imaging: US of the abdomen and regional nodes for stages I–IIA	100% (1/1)	N/A	National Paediatric Melanoma Guidelines
Imaging: US of the abdomen and regional nodes + CT of the chest for stage IIB	33% (1/3)	No specific guidance or consensus at the time of diagnosis	National Paediatric Melanoma Guidelines
Imaging: US of the abdomen and regional nodes + CT/MRI of the whole body for stage IIC and above	75% (3/4), CT/MRI of the whole body	No specific guidance or consensus at the time of diagnosis	National Paediatric Melanoma Guidelines
Wide excision margin 1 cm for BT < 1 mm	N/A	N/A	As per adult guidelines notwithstanding a smaller surface area in children
Wide excision margin 1–2 cm for BT 1–2 mm	100% (1/1)	N/A	As per adult guidelines notwithstanding a smaller surface area in children
Wide excision margin 2 cm for BT > 2 mm	89% (7/8)	Guidance changed during the period of the study, from 2010 to 2015	As per adult guidelines notwithstanding a smaller surface area in children
No specific guidance regarding completion of lymphadenectomy	100% (2/2)	N/A	For regional control for palpable disease
Discussion regarding immunotherapy management with adult melanoma oncology specialists	100% (3/3)	N/A	National Paediatric Melanoma Guidelines

The gold standard biopsy technique used for suspected adult melanoma is an excision biopsy with a 2 mm peripheral margin. This allows for an accurate BT assessment to be made to guide subsequent WLE, need for SLNB and imaging. In our study, only six patients (75%) underwent excision biopsies, with two patients undergoing curettage and cautery impacting on diagnostic accuracy. This is because curettage and shave biopsies interfere with accurate assessment of BT and should therefore only be used for lesions with a low index of suspicion [4]. Due to the diagnostic uncertainty and rarity of paediatric melanoma, curettage and cautery may also delay specimen analysis, as it is unlikely to be sent off urgently or be prioritised for analysis by the pathology team. Furthermore, the presence of atypical pathological characteristics in paediatric melanoma makes pathological analysis challenging, thereby further contributing to a delay in definitive diagnosis [10].

This study demonstrates full compliance with the main recommendation from EXPeRT that all paediatric melanoma patients are discussed in an MDT with paediatric oncologists and experienced dermatopathologists. Given the paucity of first-level evidence for paediatric melanoma management, data supporting the use of treatments is largely extrapolated from adult melanoma guidelines. This may explain the greater variability in paediatric melanoma management compared with adult management. Furthermore, the definition of the term ‘paediatric’ varies from study to study, referring to patients < 18 years in some studies and < 20 years in others [5,6,20]. Our population only included patients up to 15 years old due to local arrangements directing referrals for patients above this age threshold toward adult services. Whilst being a referral centre may have contributed to a selection bias, it is more likely that our data is an underestimate of the incidence of paediatric melanoma in the local population, given that the risk of melanoma between 15 and 20 years is much greater than that < 15 years [13].

Despite SLNB being recommended for lesions with a BT of greater than 0.8 mm, it was not performed for any patient in this study, which may be due to the difficult clinical and ethical challenges in paediatric patients. While SLNB may provide clinical information about locoregional spread, it presents an ethical dilemma due to the lack of robust evidence for immunotherapy or completion lymphadenectomy in the paediatric population, and therefore, the patient risks potentially significant morbidity, including lymphoedema and anaphylaxis, with possibly nothing to gain in terms of subsequent treatment. Clinical practice may differ in other countries, as exemplified by a single-centre US study examining the results of 33 paediatric melanoma patients, all of whom underwent SLNB [20]. The authors found that 58% (19/33) had a positive SLNB, with 53% (10/19) undergoing completion lymphadenectomy, but this did not affect the overall melanoma-specific survival [20]. These results were consistent with the much larger adult SLNB studies, MSLT-1 and MSLT-2 [21,22], despite their limited statistical significance. In our study, only one patient underwent adjuvant therapy off license for metastatic and recurrent disease, which differs greatly from the proportion of patients undergoing adjuvant therapy, amongst other studies. In the single-centre US study, 89% (17/19) of the patients with a positive SLNB underwent adjuvant therapy [20], possibly due to different health, economics and medical culture, rather than robust clinical evidence.

Wide excision margins were variable and may reflect the change in UK British Association of Dermatology Guidelines from 2010 to 2015, which replaced recommendations for excision margins of 3 cm with 2 cm following a BT of greater than 2.0 mm [23,24]. In our series, four cases were diagnosed after 2015 and were correspondingly treated with smaller excision margins. Imaging recommendations had a variable compliance of between 33% (3/8) and 100% (8/8), depending on the clinical stage. Often, initial imaging was carried out in most patients, with follow-up and surveillance imaging conducted on a more variable schedule. This is consistent with other literature where variable imaging regimes have been suggested [20].

This study has a variable follow-up period ranging from three months to over five years. Most often, patients at this tertiary centre would be referred back to non-tertiary local hospitals after an initial period of follow-up. The variability in follow-up times and procedure is consistent with other studies across the literature and may reflect the paucity of robust data, as well as variable resources and healthcare systems.

The sample size of this study is very small despite covering a period of 20 years in a major UK tertiary referral centre responsible for a population of 90,000 children. Whilst this reflects the rarity of paediatric melanoma, it also affects the statistical power of the study, and therefore, it is difficult to draw any firm conclusions from the study. Nonetheless, our data elucidated some interesting observations that appear consistent with literature worldwide. These include the wide excision margin, SLNB, treatment after SLNB, treatment of recurrence, imaging and follow-up pathways [10,12,20]. Finally, it is important to note that the EXPeRT and NCCN recommendations were written in 2017 and 2021, respectively, meaning that many of the patients in our study were treated before the recommendations were published. Furthermore, there is heterogeneity between these guidelines in terms of scope, age range covered and aims, which makes comparisons and generalisability more difficult. A national or international paediatric melanoma registry may provide an excellent foundation for more robust data analysis, early identification of emergency trends and standardisation of care. For example, the National Cancer Registration and Analysis Service (NCRAS) identified that craniopharyngiomas and skull base sarcomas benefited from centralisation of care, which in turn improved survival and reduced morbidity [25].

## Conclusions

This study shows excellent compliance with the recommendation from EXPeRT that all patients should be discussed in an MDT involving experts in dealing with adult melanoma and experienced dermatopathologists. The other recommendations have much greater variability in terms of compliance, which may reflect the rarity of paediatric melanoma. The clinical management of children and adolescents with melanoma remains challenging. The major issues concern the centralisation of care and access to clinical trials and new drugs. The current issues regarding diagnosis, excision margins, SLNB, adjuvant treatment, treatment for recurrence, imaging and follow-up require a consensus approach based on the current available best worldwide evidence. Future studies should include multicentre trials to provide higher-quality evidence to manage this challenging paediatric malignancy.

We would recommend that standardised proformas for collecting data, based on these European and American guidelines for such rare cancers, are used to ensure a more consistent approach amongst units, and to facilitate more comprehensive audit and research data. Furthermore, the National Paediatric Melanoma Specific Guidelines produced by a consensus approach may be helpful in the interim to standardise care until randomised clinical trials can ascertain more robust evidence-based practice.
